# Giant left atrial myxoma mimicking severe mitral valve stenosis and severe pulmonary hypertension

**DOI:** 10.1186/1755-7682-6-13

**Published:** 2013-04-19

**Authors:** Najat N Mouine, Ilyass I Asfalou, Maha M Raissouni, Aatif A Benyass, El Mehdi E Zbir

**Affiliations:** 1 Cardiology department, Mohamed V Military Hospital, Mohamed V University Souissi, Rabat, 10012, Morocco

## Abstract

Myxoma is the most common primary tumor of the heart and can arise in any of the cardiac chambers. This paper reports A 50 -year-old woman without medical history and any cardiovascular risk factors was hospitalized for exertional dyspnea and palpitations from three months and signifiant weight loss. Transthoracic echocardiogram showed a giant left atrial myxoma mobile confined to the left atrium in systole, in diastole the tumor was seen prolapsing across the mitral valve into the left ventricle and partially obstructing it and causing severe functional mitral stenosis with a mean gradient of 21,3 mmHg. Severe pulmonary hypertension was confirmed by Doppler PAPs =137 mmHg. The patient was scheduled for cardiac surgery with good outcome.

## Introduction

Myxoma is the most common primary tumor of the heart and can arise in any of the cardiac chambers, although the left atrium is the most commonly involved (75%) [1,2]. The classic triad of myxoma clinical presentation is characterized by intracardiac obstruction, embolisms and constitutional symptoms with fever, weight loss, or symptoms resembling connective tissue disease [1-3].

## Case report

A 50-year-old woman patient without medical history and any cardiovascular risk factors was hospitalized for exertional dyspnea and palpitations from three months and signifiant weight loss. She related worsening of symptoms in the last week before admission.

On admission, she was in the poor condition, cachectic, weighing 45 kg for a height of 1, 65 m and a BMI 17 kg/ m2, blood pressure was 100/60 mm Hg, heart rate was regular 120/min, respiratory rate was 29 breaths/ min and she was afebrile. Oxygen saturation was 98%. Physical examination revealed bilateral lung crepitations. A full blood cell count showed a mild normocytic anemia (hemoglobin = 11.5 g/dl). A chest radiograph showed left atrial enlargement and data of left cardiac failure. The electrocardiogram showed sinusal tachycardia.

**Figure 1:**
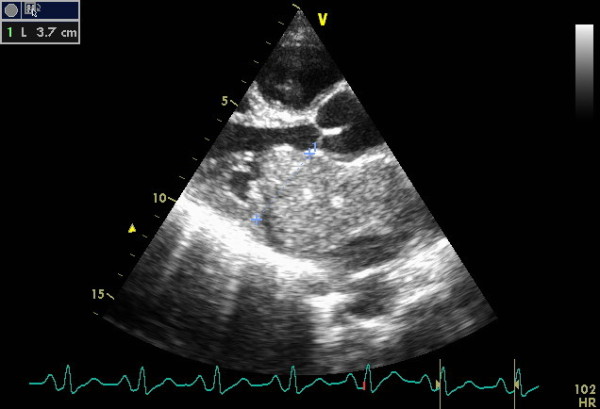
Transthoracic echocardiography long-axis view shows a giant left atrial myxoma fills almost the entire left atrium.

**Figure 2:**
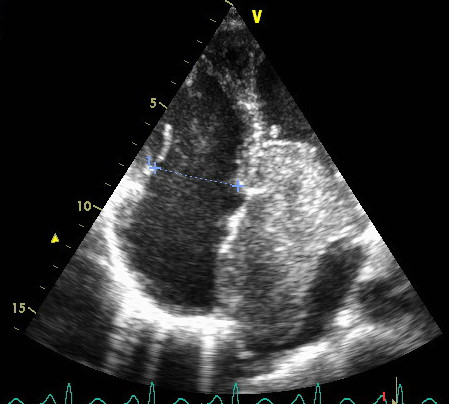
Transthoracic echocardiography 4 cavity view shows a giant left atrial myxoma fills almost the entire left ventricular at diastole.

Transthoracic echocardiography performed in emergency showed a giant left atrial mobile mass (72× 53 mm) attached to the interatrial septum and confined to the left atrium in systole (Figure 1). In diastole (Figure 2) the tumor was seen prolapsing across the mitral valve into the left ventricle and partially obstructing it and causing severe functional mitral stenosis with a mean gradient of 21,3 mmHg. A mild mitral regurgitation was also found. Severe pulmonary hypertension was confirmed by Doppler PAPs =137 mmHg. The patient was scheduled for cardiac surgery. During the operation, the tumor was excised through a sternotomy from a transseptal approach with a small portion of the atrial wall and the histopathological analysis found atrial myxoma. The clinical course was uncomplicated and the patient was discharged after three weeks with good outcome.

## Discussion

Primary cardiac tumors occur infrequently with an incidence of 0.0017–0.19% in autopsy series performed in non-selected populations [1-3]. Myxomas are more common among women and occur much more frequently between the ages of 30–60 years [1-3]. Left atrial myxomas become symptomatic when they obstruct the mitral valve, embolize peripherally or cause systemic effects. The size, location and mobility of myxoma termine the seriousness of mitral valve obstruction. The smptoms vary from dyspnea due to heart failure or syncope to sudden death due complete mitral obstruction [2-5]. Up to more than a half of left atrial myxomas show obstructive symptoms [3], but only in 10% of patients will it cause severe mitral stenosis [4-6]. In our case, the giant myxoma occupied almost the entire dilated left atrial cavity causing a severe mitral valve stenosis and severe pulmonary hypertension. In young patient with congestive heart failure can masquerade as mitral valve disease.

The early echocardiography exam plays a pivotal role in the diagnosis and clinical management of these patients. In this regard, a recent large series of left atrial myxoma emphasizes this topic because the presence of cardiac signs pradoxically could increase the diagnosis delay, probably due to the belief that clinical symptoms are explained by another more common disease, like hypertensive cardiomyopathy or ischemic heart disease [7,8]. The surgical treatment is usually curative, like our case, where the patient had cardiac surgery with good outcome. Recurrences are exceptional, especially observed in the family forms.

## Consent

A written informed consent was obtained from the patient for the publication of this paper and the accompanying images.

## Competing interest

The authors declare that they have no competing interest.

## Authors’ contributions

IA participated in the sequence alignment and drafted the manuscript. MR participated in the sequence alignment and drafted the manuscript. AB participated in the sequence alignment and drafted the manuscript. EZ participated in the sequence alignment and drafted the manuscript. All authors read and approved the final manuscript.
